# An unusual presentation of breast tuberculosis: A case report

**DOI:** 10.1002/ccr3.3500

**Published:** 2020-11-11

**Authors:** Giovanna Rizzo, Francesco Colli, Paolino De Marco, Antonella La Brocca, Gabriella Militello, Gaspare. Gulotta

**Affiliations:** ^1^ Unit of General and Emergency Surgery Department of Surgical, Oncological and Oral Sciences University Hospital P. Giaccone University of Palermo Palermo Italy; ^2^ Department of General Surgery Nuovo Ospedale Civile Sant'Agostino Estense di Baggiovara Baggiovara Italy

**Keywords:** breast tuberculosis, case report, differential diagnosis, primary breast tuberculosis

## Abstract

Primary breast tuberculosis is an uncommon disease, especially in nonendemic areas. Its presentation could mimic a cancer or an abscess, but this entity must be considered for differential diagnosis in women coming from endemic countries.

## Graphical abstract

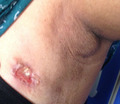

## 1 BACKGROUND

Tuberculosis (TB) disease mainly affects lungs but may also have extrapulmonary localizations. Primary breast tuberculosis (PBT) is defined as a tuberculosis localized only in the breast, in a patient with no history of pulmonary TB. PBT results very rare and represents 0.06%‐0.1% of all tuberculosis localizations.^1^, ^2^ In 1829, Sir Astley Cooper recorded the first case of breast tuberculosis that he reported as “scrofulous swelling of the bosom”.^3^ This disease usually affects young, multiparous, breastfeeding women and its incidence is higher in TB endemic countries, like the Indian subcontinent, where PBT’s incidence is around 4%.^4^

## 2 CASE PRESENTATION

**FIGURE 1:**
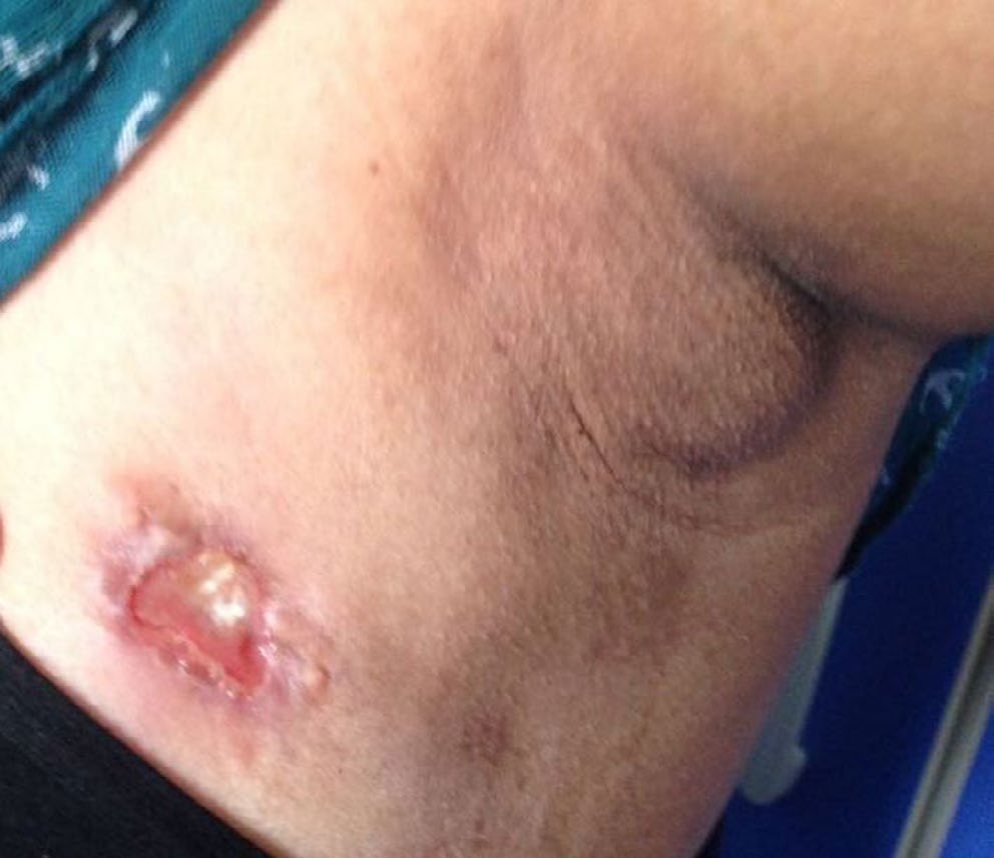
Breast lesion with axillary lymphadenopathy

**FIGURE 2:**
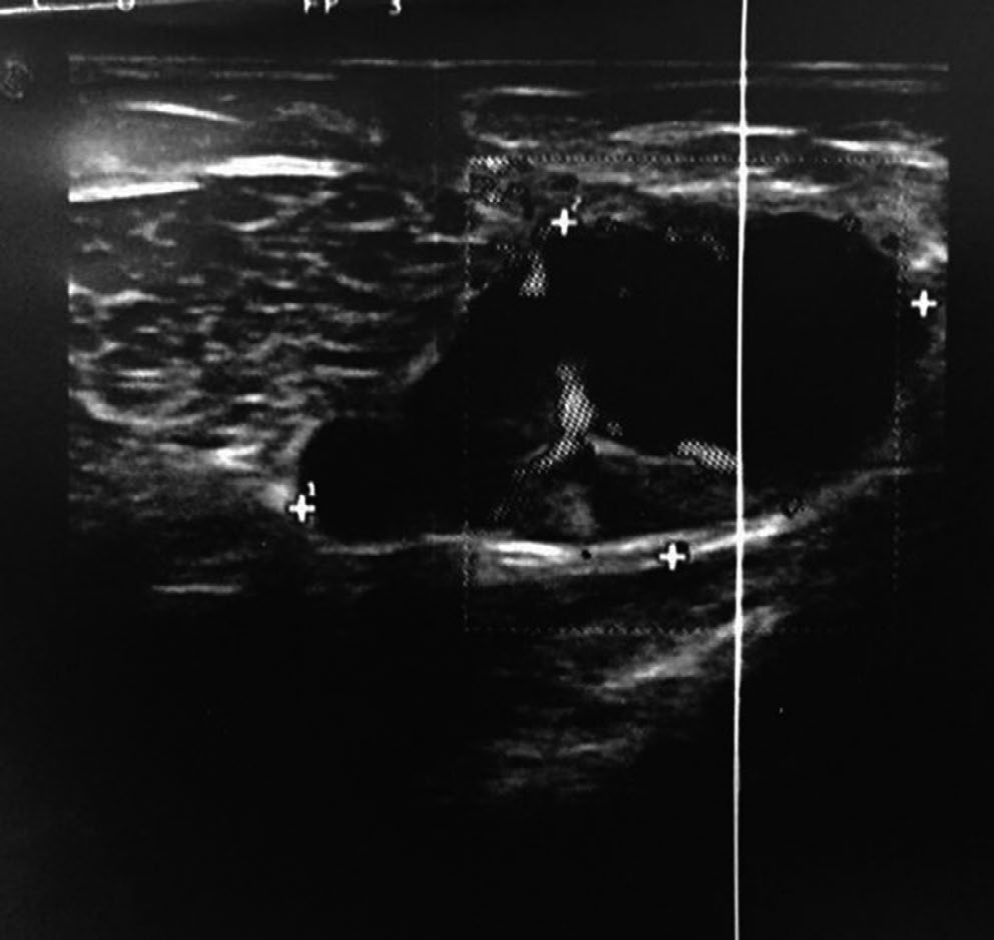
Etg image of the lesion

A 19‐year‐old woman from Bangladesh was admitted to our Surgery Department from Emergency Room, presenting a painful lump in the upper outer quadrant of the left breast. The lesion measured about 2 cm in size, ulcerated, leaking pus‐like material (Figure 1). Ipsilateral axillary lymphadenopathy was associated, without cutaneous fistula. According to the patient, it was a longstanding lesion, but she refused any previous physical examination due to religious matter. She denied history of fever, smoking, breastfeeding, use of contraceptive pills, injury, or exposure to radiation of the breast. A pus‐like sample was collected through needle aspiration for microbiological and biomolecular examinations. Breast ultrasound integration found the presence of a cystic lesion of 35 × 19 mm in size, with posterior wall reinforcement, peri‐lesional hypervascularization, and reactive lymphadenopathy (Figure 2). A chest X‐ray confirmed absence of tuberculosis pulmonary's focuses. Mammography was not performed due to the young age of the patient. To exclude breast cancer, a tru‐cut biopsy was performed. The histopathological report highlighted granulomatous inflammation with caseous necrosis, rod‐shaped forms positive to Ziehl‐Neelsen staining for acid‐fast bacilli (AFB), identified as Mycobacterium tuberculosis. Pus culture turned out to be negative but PCR analysis was positive for M. tuberculosis complex. So the patient was referred to Tuberculosis Diagnosis and Treatment Centre for antituberculous therapy, achieving complete remission. No surgery was performed. During the follow‐up of 2 years, there was no evidence of new disease or relapse.

## 3 DISCUSSION

Breast tuberculosis can mimic carcinoma, and it can be mistaken for a pyogenic breast abscess. For this reason, Gon et al named it the “great masquerader”.^5^ A painful lump is the most common clinical presentation, often located in the central or upper outer quadrant of the breast, followed by inflammation, abscess formation, skin ulceration, or diffuse mastitis.^6^ It is usually frequent in multiparous and breastfeeding women, although our patient was nulliparous, with no fever. In clinical practice, fever is a symptom that helps to differentiate mycobacterial infection from malignancy.

In a recent study conducted in India, an endemic area for TB disease, eight patients with different PBT lesions were analyzed. At the physical examination, they presented lesions including firm nodules and skin erythematous induration, with clinical impression of an abscess, or fibroadenoma, or cancer, highlighting PBT diagnostic challenges.^7^

Regarding the radiological aspect, no specific mammographic signs of mammary tuberculosis are individualized. At ultrasonography often appears as a hypoechoic/anechoic, heterogeneous image well or poorly limited with minimal posterior reinforcement.^8^ For this reason, PBT goes into differential diagnosis with the cysts and with all the other mammary lesions. Computed tomography and nuclear magnetic resonance are used to evaluate the extension of the lesion toward the thoracic wall. The gold standard for the diagnosis of breast tuberculosis is the detection of M. tuberculosis by Ziehl‐Neelsen staining or by microbiological culture. Fine needle aspiration cytology instead it is unable to identify the presence of the etiologic agent, but it contributes by detecting the presence of epithelioid cell granulomas and necrosis. PCR is highly sensitive to breast TB diagnosis, although rarely used since it is recommended in cases with negative culture or for differential diagnosis among other forms of granulomatous mastitis.^9^, ^10^, ^11^

## 4 CONCLUSIONS

Primary breast tuberculosis should always be suspected in patients from endemic areas, especially in young women presenting with a breast abscess. In these cases, it is necessary to perform a microbiological examination. The focus on the diagnosis of PBT is very important nowadays due to globalization and increasing immigration from endemic countries.

## CONFLICT OF INTEREST

None declared. The data that support the findings of this study are available on request from the corresponding author.

## AUTHOR CONTRIBUTIONS

GR: involved in manuscript preparation and data acquisition. FC: involved in manuscript review. PDM: involved in data acquisition. ALB: involved in manuscript review. GM: involved in clinical management. GG: involved in conception and design of the work.

## ETHICAL APPROVAL

Informed consent was obtained from the patient.
